# LightFIG: simplifying and powering feature interactions via graph for recommendation

**DOI:** 10.7717/peerj-cs.1019

**Published:** 2022-06-20

**Authors:** Weiqiang Di

**Affiliations:** School of Computer and Information Technology, Beijing Jiaotong University, Beijing, Beijing, China

**Keywords:** Recommender systems, Attribute interactions, Graph neural networks, Collaborative filtering

## Abstract

The attributes of users and items contain key information for recommendation. The latest advances demonstrate that better representations can be learned by performing graph convolutions on attribute graph of the user-item pair. Recently proposed models construct graphs that not only connect edges between user attributes and item attributes, but also within user (item) attributes. However, to determine whether a user is interested in an item, the relationships between user attributes and item attributes are what we really need to mine. In many cases, due to the low correlation between relationships within user (item) attributes and preference of the user, the artificially connected edges within user (item) attributes contribute little to the recommendation. Even worse, including them will not only drastically increase the training time, but may also introduce noise and lead to degraded performance. In addition, the use of the optimizer is also relatively simple. One single optimizer is the default configuration for recommendation models. This may not be the best way to exploit it in many cases however. To solve these problems, we propose an enhanced model named LightFIG in this work. The key idea of LightFIG is twofold: First, we simplify the construction of attribute graph which focuses on mining relationships cross user attributes and item attributes, not between user (item) attributes. Second, we propose the idea of relay optimization, which employs two different optimizers to continuously optimize model parameters. Comprehensive experiments on three public datasets demonstrate the effectiveness of our proposed model.

## Introduction

With the explosive growth of online information, recommender systems have become indispensable tools for many businesses like online e-commerce platforms (*e.g.*, Amazon and Taobao) and information streaming medias (*e.g.*, YouTube and TikTok). Users rely on recommender systems to alleviate information overload and find what they are interested in from the huge pool of items. In common recommendation systems, users’ behavior sequence, *e.g.*, clicks and purchases, are leveraged to predict whether a user will show interest to an item. Collaborative filtering (CF) is a fundamental technique that can produce effective recommendations from implicit feedback. By assuming users that share common interacted items tend to have similar interests, CF predicts users’ preferences through similarity patterns found across users with user profiles and item attributes as input features. Using raw features directly can hardly get satisfying results; thus, feature interactions modeling continues to attract lots of attention from both academia and industry (Guo et al., 2017; Qu et al., 2016; Song et al., 2019; Beutel et al., 2018; Cheng et al., 2016; Naumov et al., 2019). The common paradigm for these models is to learn latent embeddings to represent input features, and then transformed into more abstract fixed-length vectors *via* feature interactions, finally fed into fully connected layers to perform prediction.

Effective feature interactions are critical to the success of many methods, which provide supernumerary interaction information beyond individual features. For instance, the combination of features “gender” and “age” is more informative than either one of them when recommending movies. Traditional methods implicitly capture the collaborative signals which use recorded interactions as the supervised signals. Factorization machine (FM) (Rendle, 2010) embeds each feature into a vector representation, and constructs pairwise feature interactions *via* the inner product. Due to its simplicity and effectiveness, Attentional Factorization Machine (AFM) (Xiao et al., 2017) further extends FM by capturing the weight of each feature interactions using attention mechanism. Neural Factorization Machine (NFM) (He & Chua, 2017) then captures the complex and nonlinear relationships between users and items by using multi-layer perceptron (MLP). People generally consider MLP as a universal function approximator, which means almost any form of feature interactions can be learnt (Mhaskar, 1996). However, recent study (Beutel et al., 2018) found that deep neural networks (DNNs) are inefficient to simulate even second-order or third-order feature interactions. Wide&Deep (Cheng et al., 2016) combines the strength of both the linear model and MLP. DeepFM (Guo et al., 2017) replaces the “wide” module in Wide&Deep with a factorization machine. Due to the limited expressiveness of MLP, AutoInt (Song et al., 2019) uses a self-attentive neural network to learn high-order feature interactions. Despite their effectiveness, most existing methods do not explicitly consider the attribute interactions, which may limit models’ ability in capturing the crucial collaborative signals. Different from traditional models that only implicitly mine the collaborative signals, graph neural networks (GNN) (Gori, Monfardini & Scarselli, 2005) naturally and explicitly encode them *via* topological structure and perform information propagation on the graph to learn the user and item representations (Zhang & Chen, 2019; Huang et al., 2019; Wang et al., 2019; Wang et al., 2020; Sun et al., 2020). In this scenario, the behavior sequence recorded in datasets can be represented by a bipartite graph between user and item nodes, with observed interactions represented by edges. However, they only model interaction behaviors into the graph structure. An issue in directly using GNN on the bipartite graph is that such graph structure may not be sufficient enough for learning user/item representations, especially when we have attribute information. Fi-GNN (Li et al., 2019b), *L*_0_-SIGN (Su et al., 2021a) and GMCF (Su et al., 2021b) are then proposed to construct graphs using attributes of the user-item interaction pair to model relationships among attributes and enhance recommendation.

Although they have shown promising results, we argue that the graph construction in their models are rather heavy and burdensome. They build a complete attribute graph for each user-item pair, where each attribute belonging to the user or item is a node and every pair of distinct nodes is connected by a unique edge. Since both users and items are fully characterized by their attributes, if a user shows preference for an item, some attributes of the user must have strong relationships with some attributes of the item. For example, we can access three user attributes in a book recommendation task: ID, nationality and age. The information in item attributes are richer such as ISBN, language, category, publication date, author, title, etc. If the nationality of a user is China, the recommended books for that one are best written in Chinese. If this user is a teenager, he/she is likely to prefer some storybooks to other genres. We can see that for each useful attribute belonging to the user, the suitable attributes of the recommended item should be restricted accordingly. Such kinds of correlations crossing user attributes and item attributes directly affect the effectiveness of recommendation which we should focus on capturing, not the internal relationships within user (item) attributes themselves. However, they are forced to be related *via* a complete graph in current models since there is no supervision signal in the attributes level. These massively increased redundant edges not only greatly increase the computational complexity, and may even have a negative impact on the model training which will be illustrated in the ablation study. Next we look at another aspect that can be improved. In the recommendation field, the usual practice is to select a commonly used optimizer such as Adam and then configure the required parameters. This single-optimizer mode has long been followed with good results. However, sometimes the commonly used optimizers do not fit the characteristics of experimental datasets, leading to a quickly ended optimization process. At this moment, it is worth combining multiple optimizers to co-optimize model parameters since different optimizers have their own characteristics and advantages. They can collaborate to find a better optimization path. While it is not considered by the mainstream before.

In this article, we propose a novel model LightFIG, which is designed with two considerations to address the above two challenges in existing methods. Specifically, we construct a simplified graph which gets rid of traditionally connected edges between the user (item) attributes themselves and improve the message propagation mechanism. Further more, we evolve the traditional single-optimizer mode to the dual-optimizer mode, which divides the optimization of model parameters into two stages. Each optimizer is responsible for one stage, and two stages relay the optimization process continuously.

To summarize, The main contributions of the article are three fold:

• We highlight the limitation of the graph construction scheme in previous models and focus on mining relationships only between user attributes and item attributes, not within themselves.
• To the best of our knowledge, our work is the first one to introduce the dual-optimizer mode to relay optimize the model parameters, which is useful in the scenario where the optimizer converges too quickly.
• We perform extensive experiments on three public datasets, demonstrating significant improvements of our model over state-of-the-art methods. The necessity of the two kinds of improvements is verified empirically.

## Related Work

We reviewed existing work on attribute-aware CF, graph-based CF and optimization methods, which are most relevant to our article.

### Attribute-aware recommendation

Extensive studies on CF recommendation have been carried out and achieved great success. Attributes of users/items are important information for preferences, and their proper use plays a central role in improving the recommendation performance. It is essential to extract informative representations from the user-item interactions and attributes. Embedding techniques have been widely used to project features from high-dimensional sparse vectors to low-dimensional dense vectors. Factorization machine (FM) (Rendle, 2010) is an early popular model which projects both users and items into a low latent space and utilizes inner product to learn pairwise interactions between every two attributes. It gained a huge impact due to its simplicity and effectiveness and is followed by many work. However, FM can not obtain the complex interactions of different features. AFM (Xiao et al., 2017) further strengthened FM by learning the influence of each cross feature using the attention mechanism. The linearity of inner product makes it insufficient to learn the complex and nonlinear relationships between users and items. To make up for this shortcoming, NFM (He & Chua, 2017), Wide&Deep (Cheng et al., 2016) and DeepFM (Guo et al., 2017) are then proposed to use various kinds of linear and nonlinear multi-layer perceptron (MLP) to enhance their capability. Instead of feature interactions generated by a single model, multi-interaction ensembles are employed to take advantages of different models. In Wide&Deep, it combines LR and MLP and in DeepFM, it combines FM and MLP. For tasks with high-order features, MLP is not sufficiently expressive to capture such information. AutoInt (Song et al., 2019) takes a different approach and makes use of the latest techniques, self-attention mechanism and residual networks, to generate non-linear features. Despite good performance, we argue that the above works are insufficient to yield optimal embeddings for CF, since the collaborative signals are only implicitly learned and forgoing their relationships. Hence, we pay attention to mining relationships among attributes in this work.

### Graph neural networks for recommendation

Another relevant research line is to leverage graph neural networks(GNN) (Gori, Monfardini & Scarselli, 2005; Scarselli et al., 2008), which consider information from the perspective of graphs for recommendation since many datasets have a graph structure essentially (Bruna et al., 2013; Henaff, Bruna & LeCun, 2015). The core operation in GNN is the embedding propagation mechanism, which aggregates the representation of neighbor nodes to update the central node’s representation. GraphSAGE (Hamilton, Ying & Leskovec, 2017) proposes three types of aggregators: LSTM aggregator, Pooling aggregator and Mean aggregator. The attention mechanism is introduced in the propagation process at the graph attention network (GAT) (Veličković et al., 2017). GNN models have been widely adopted in many fields for their outstanding representation ability (Gong & Cheng, 2019; Zhang & Chen, 2018; Xu et al., 2019). The advantage of GNN is that it provides a powerful mechanism to explore multi-hop relationships which have been proven beneficial for recommendation tasks (Wang et al., 2019; Ying et al., 2018). Motivated by the strength of GNN, some works adapt GNN to the user-item interaction graph to better capture the collaborative signals, where users and items act as nodes while an interaction like purchase or click constitutes an edge between them Huang et al. (2019); Wang et al. (2020); Sun et al. (2020). However, they only convert users’ behaviors into the graph. Fi-GNN (Li et al., 2019b), *L*_0_-SIGN (Su et al., 2021a) and GMCF (Su et al., 2021b) are models proposed to explore the attribute graph of the user-item pair to learn relationships among attributes and improve recommendation. However, in these models of attribute graph, the construction of graphs is still dominated by the complete graph, where every pair of distinct nodes are linked. This way is rather heavy and burdensome. In the attribute graph, relationships within user (item) attributes themselves are not important for the recommendation task in the context of our problem. The preservation of these edges will not only greatly increase the overhead of training time, but may also introduce noise and lead to performance degradation. In addition, in the message propagation mechanism of GNN, they employ element-wise multiplication between every two neighbors to model the attribute relationships. However, more effective feature crosses can be exploited to provide additional interaction information. In this article, we propose to construct a simplified attribute graph which only links nodes between the user attributes and item attributes. We then improve the message propagation mechanism by designing multiple informative feature crosses, such as concatenation, element-wise product and element-wise addition, and then integrating them. Finally, following the residual connections of Resnet (He et al., 2016), we propose a graph-level gating layer, which integrates the original embedding and updated representation of users/items after graph convolutions using gating mechanism, to help with training.

### Optimization methods

The goal of many machine learning methods is to update model parameters by optimizing their objective functions. An iterative process is involved which applies incremental modifications to the trainable parameters. Training a large deep neural network can be painfully slow. Using a faster optimizer than the regular stochastic gradient descent (SGD) can get huge speed boost. There are some improved algorithms to the primitive gradient descent. One method of speeding up training per-dimension is the Momentum method (Rumelhart, Hinton & Williams, 1986). The Momentum’s key operation is to accelerate progress along dimensions in which gradient consistently point in the same direction and to slow progress along dimensions where the sign of the gradient continues to change. AdaGrad (Duchi, Hazan & Singer, 2011) performs well for sparse gradients on large scale learning tasks, which uses only first order information but has some properties of second order methods. RMSprop (Tieleman & Hinton, 2012) is an optimization algorithm that generates its parameter updates using a momentum on the rescaled gradient. It has a close relation with Adam (Kingma & Ba, 2015), which will be stated later. In particular, RMSprop is essentially a special case of Adam with *β*_1_ = 0. Adam, A prominent first-order optimization algorithm, is very popular and often used as the default optimizer. It is derived from the above two well-behaved optimizers: an exponential moving mean value of past gradients and squared gradients are taken as done in AdaGrad and RMSprop respectively. The current recommendation models basically use a single Adam optimizer to optimize network parameters by default (Li et al., 2019b; Su et al., 2021a; Su et al., 2021b; Huang et al., 2019; Wang et al., 2020; Sun et al., 2020). This certainly reflects people’s trust in it, but more diverse ways of using optimizers still need to be developed to deal with some challenging scenarios. For example, when commonly used optimizers stop quickly due to the mismatched characteristics on some datasets, combining the strengths of multiple optimizers to co-optimize is an attractive and profitable option, which may find a better way to optimize parameters. This is exactly what we propose in this article.

## Methodology

In this section, we introduce the design of the proposed model LightFIG. The recommendation problem is first formulated. We then elaborate on the details of LightFIG.

### Problem definition

We formulate the recommendation task with necessary notations here. Let *U* and *V* denote the sets of users and items, and *Y*_*M*×*N*_ denotes the interaction matrix where *M* and *N* are the number of users and items. Here, an observed interaction *y*_*uv*_ = 1 in *Y* means user *u* has interacted with item *v* in history, otherwise *y*_*uv*_ = 0. There are a set of *J* user attributes $A= \left\{ {a}_{1},{a}_{2},\ldots ,{a}_{J} \right\} $ and *K* item attributes $B= \left\{ {b}_{1},{b}_{2},\ldots ,{b}_{K} \right\} $. Each user and item is associated with a list of attributes *A*_*u*_ ⊂ *A* and *B*_*v*_ ⊂ *B*. It should be noted here that the identification index of each user/item is also treated as one of the attributes. After concatenating all the features, one input example can be represented as:

(1) \begin{eqnarray*}x=[{A}_{u},{B}_{v}].\end{eqnarray*}

The purpose of the task is to design a predictive model so that given an input *x*, the model can output the probability *y* that the target user *u* interacts with the candidate item *v*.

### Overall architecture

**Figure 1:**
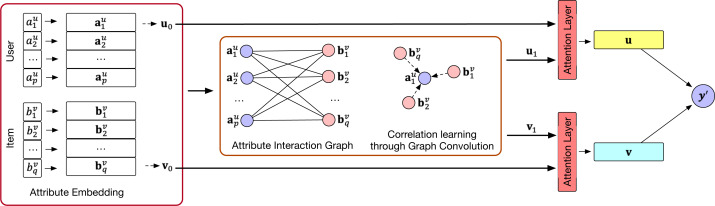
An overview of the LightFIG model.

We summarize the general workflow of the proposed LightFIG in Fig. 1, which roughly includes three parts: (1) the embedding layer that projects all attributes into a low-dimensional space; (2) the embedding propagation layer that refines embeddings by injecting connectivity relations between attributes; and (3) the prediction layer that aggregates the refined representations and outputs the matching score of a user-item pair. We next describe each component in detail.

### Embedding layer

The multi-field categorical feature *x* is usually sparse and of huge dimension. Following mainstream recommendation models, we associate each attribute with an embedding vector, resulting in a set of embeddings to describe the input user and item, respectively. For example, the embedding vector of a *m*-field input can be obtained:

(2) \begin{eqnarray*}\mathbf{E}= \left[ {\mathbf{e}}_{1},{\mathbf{e}}_{2},{\mathbf{e}}_{3},\ldots ,{\mathbf{e}}_{m} \right] \end{eqnarray*}

where **e**_*i*_ ∈ ℝ^*d*^ denotes the embedding vector of attribute *i* and *d* is the embedding size. We include the identification index into the user and item attributes for that it helps to distinguish users (items) when their other features are the same.

### Graph convolution layer

In our model, the embedding vectors were refined by propagating them on the attribute graph. This can lead to more effective representations as it will augment embeddings with explicit collaborative signals by aggregating a node’s neighbors. In general, a user’s preference is reflected among the interacted items. As a user or item is completely characterized by their attributes, we can reason that a strong co-occurrence pattern exists between some attributes of the user and some attributes of the item if the user is interested in that item.

#### Graph construction

Graph structure is essential for the scope and type of information to propagate. Given the attributes of the user and item, we then consider how to construct a graph connecting them. We represented each input of multi-field features as an attribute graph, where each node corresponds to an attribute. The attribute can be in multiple users or items, serving as a bridge to improve its representation. The construction of edges is critical. Unfortunately, there is no supervision signal to tell us which attributes are strongly correlated and should be linked. In previous models, a complete graph is constructed among the attributes included in each interaction sample, where any two attributes are connected by an edge. However, we propose to link nodes only between user attributes and item attributes and there is no edge within the user (item) attributes. This is because in many cases, whether a user is interested in an item depends on the matching degrees between the user attributes and the item attributes, not the relationships within user (item) attributes themselves. Formally, the input interaction **x** can be represented by an undirected attribute graph $G= \left( V,L \right) $. The nodes in *V* consist of the user attributes *a*_*i*_ ∈ *A*_*u*_ and item attributes *b*_*j*_ ∈ *B*_*v*_. The edges in *L* are denoted as ${e}_{st}= \left( s,t \right) $ with *s* ∈ *A*_*u*_ and *t* ∈ *B*_*v*_ (or *s* ∈ *B*_*v*_ and *t* ∈ *A*_*u*_ alternatively for the sake of undirected edges). The attribute graph establishes link relations to better mine correlations and co-occurrence between attributes.

#### Embedding propagation layer

With the attribute graph at hand, it is now time to improve feature embeddings with the graph convolution. We will build upon the message-passing mechanism of common GNNs and make certain changes. The graph convolution on the attribute graph is formulated as follows:

(3) \begin{eqnarray*} & {\mathbf{a}}_{u}^{(l+1)}=\sum _{v\in {N}_{u}}g({\mathbf{a}}_{u}^{(l)},{\mathbf{b}}_{v}^{(l)}) & & {\mathbf{b}}_{v}^{(l+1)}=\sum _{u\in {N}_{v}}g({\mathbf{b}}_{v}^{(l)},{\mathbf{a}}_{u}^{(l)})\end{eqnarray*}

where ${\mathbf{a}}_{u}^{(l)}$ and ${\mathbf{b}}_{v}^{(l)}$ denote the attribute representation of the user and item at the *l*-th graph convolution layer, and *g*(⋅) is a self-defined function. One layer of graph convolution will explore first-order relationships between linked attributes and co-interacted attributes for second-order connectivity. More layers can be stacked to mine the high-order correlations.

##### Message Propagation

In graph convolutional networks, the representation for each node is updated by integrating the embedding of the node itself with the node embeddings in its local neighborhood. The message propagation layer receives the information transferred from directly connected nodes to prepare for the later neighborhood aggregation. For a pair of linked nodes (*s*, *t*), we define the message transferred from *t* to *s* as:

(4) \begin{eqnarray*}{\mathbf{m}}_{st}=g({\mathbf{e}}_{t},{\mathbf{e}}_{s})\end{eqnarray*}

where **m**_*st*_ is the propagated message from node *t* to *s*.*g*(⋅) is a message propagation function, which takes representations of both the central node **e**_*s*_ and one directly connected node **e**_*t*_ as input. The propagation step is of the vital importance for graph convolution, which decides how neighbors’ information is propagated. We implement *g*(⋅) in our work as follows:

(5) \begin{eqnarray*}{\mathbf{m}}_{st}=\text{ReLU} \left( \right. ({\mathbf{e}}_{t}{|}{|}{\mathbf{e}}_{s}){\mathbf{W}}_{1}+({\mathbf{e}}_{i}\odot {\mathbf{e}}_{u}){\mathbf{W}}_{2}+({\mathbf{e}}_{i}+{\mathbf{e}}_{u}){\mathbf{W}}_{3} \left( \right. {\mathbf{W}}_{4}\end{eqnarray*}

where **W**_1_ ∈ ℝ^2*d*×*h*^, **W**_2_, **W**_3_ ∈ ℝ^*d*×*h*^, **W**_4_ ∈ ℝ^*h*×*d*^ are trainable transformation matrices to learn multiple feature crosses. *h* is the hidden size of transformation, || is the concatenation operation, ⊙ denotes the element-wise product and + represents the element-wise addition. Operation ⊙ can passes more messages from the similar attributes. The addition operation + can highlight features with large accumulated values. The concatenation operation || is more flexible and can capture the influence between features of different dimensions. These three feature crosses can effectively enrich model’s feature quality and finally lead to better performance for recommendation.

##### Neighbor aggregation

Now we need to aggregate the neighbors information of the central node. Sum-pooling is one of the most straightforward aggregation operations. We generate the updated representation of a node by aggregating its neighbors’ representations through the following way:

(6) \begin{eqnarray*}{\mathbf{m}}_{s}=\sum _{t\in {N}_{s}}{\mathbf{m}}_{st}\end{eqnarray*}

where **m**_*s*_ ∈ ℝ^*d*^ is the message passing result of node *s* and *N*_*s*_ is a set containing items that node *s* links with.

In many cases, the representation of attributes can be refined by their multi-hop neighbors, which can be captured by stacking more such graph convolution layers. The suitable layer number varies with datasets.

##### User/Item Representation

Since a user (item) is composed of multiple attributes, we use both the mean and max pooling to pool all nodes constituting them and obtain their representations. This can characterize user (item) representations from different perspectives. Formally, the pooling function is:

(7) \begin{eqnarray*} & \mathbf{u}= \frac{1}{{|}{\mathbf{A}}_{u}{|}} \sum _{i\in {\mathbf{A}}_{u}}{\mathbf{m}}_{i}+\max _{j\in {\mathbf{A}}_{u}}{\mathbf{m}}_{j} & & \mathbf{v}= \frac{1}{{|}{\mathbf{B}}_{v}{|}} \sum _{i\in {\mathbf{B}}_{v}}{\mathbf{m}}_{i}+\max _{j\in {\mathbf{B}}_{v}}{\mathbf{m}}_{j}\end{eqnarray*}

where |*A*_*u*_| is the size of set *A*_*u*_, **u** and **v** are the refined representations of the user and item respectively.

### Prediction layer

The output of the graph convolution layer encodes structural information of attributes connections into embeddings. With this information at hand, the role of the prediction layer is to output a prediction by synthesizing existing useful information.

#### Graph-level gating layer

At this stage, we can obtain two representations of the user (item). One is the original embedding representation before the graph convolution layer, which is got by applying the sum pooling on all attribute embeddings belonging to the user (item). The second is the representation **u** (**v**) learned after the graph convolution layer, which can be got by applying the same pooling method on all the updated attribute representations. We re-denote the two representations of the user (item) as **z**_0_ and **z**_1_ for notation unity, where **z**_0_, **z**_1_ ∈ ℝ^*d*^. Due to the good performance of Resnet (He et al., 2016)’s residual connections, we follow it and add the original embedding and updated representation of users/items to help with training. Slightly different from the original method, we fuse them through a gating mechanism. This is done as follows:

(8) \begin{eqnarray*} & \lambda =\sigma ((mean({\mathbf{z}}_{0}){|}{|}mean({\mathbf{z}}_{1}))\mathbf{W}) & & \mathbf{z}=\lambda [0]\cdot {\mathbf{z}}_{0}+\lambda [1]\cdot {\mathbf{z}}_{1}\end{eqnarray*}

where *mean*(**z**_0_), *mean*(**z**_1_) ∈ ℝ^1^ is the mean pooling of vectors **z**_0_ and **z**_1_, we concatenate the two pooling values and put them through a linear transformation layer with a trainable matrix **W** ∈ ℝ^2×2^.*σ* is the sigmoid function to limit the coefficients between 0 and 1.

#### User-item matching

After the integration of Eq. (8), we can get final representations of the user and item and denote them with **z**_*u*_ and **z**_*v*_ respectively. It is important to explicitly model interactions between the target user and item for preference prediction. Considering the simplicity and effectiveness of the dot product, we select it to estimate the user’s preference towards the target item as follows.

(9) \begin{eqnarray*}{y}^{{}^{{^{\prime}}}}={{\mathbf{z}}_{u}}^{\top }{\mathbf{z}}_{v}.\end{eqnarray*}

### Model training

To optimize model parameters, we opt for the binary cross-entropy loss, which has been intensively used in recommender systems. *L*_2_ regularization is also employed to prevent overfitting. Then we minimize the following objective function:

(10) \begin{eqnarray*}L=- \frac{1}{N} \sum _{j=1}^{N}({y}_{j}log({y}_{j}^{{}^{{^{\prime}}}})+(1-{y}_{j})log(1-{y}_{j}^{{}^{{^{\prime}}}}))+\lambda ({|}{|}\theta {|}{{|}}_{2})\end{eqnarray*}

where *y*_*j*_ and ${y}_{j}^{{}^{{^{\prime}}}}$ are ground truth of user clicks and estimated prediction respectively, *N* is the total number of training samples, *θ* represents all trainable parameters in our model and *λ* controls the strength of penalty.

### Dual optimizer relay mechanism

Stochastic gradient-based optimization is very important in many fields of today’s machine learning. Many problems can be transformed as the optimization of an objective requiring minimization with respect to its parameters. These objective functions can have some sources of noise such as data subsampling and dropout regularization. Efficient optimization techniques are required to overcome the noisy objective function. Adaptive optimization methods like AdaGrad (Duchi, Hazan & Singer, 2011), RMSprop (Tieleman & Hinton, 2012) and Adam (Kingma & Ba, 2015) have been proposed to learn fast with an element-wise scaling term on learning rates.

In recent recommender systems, there is little improvement in the application of optimizers. Summarizing existing models, a common process is to choose a single mainstream optimizer like Adam and set the corresponding learning rate. While each optimizer has its own unique design philosophy and advantage. For example, AdaGrad works well with sparse gradients and RMSprop has good performance in on-line and non-stationary settings. In some scenarios, The common optimizers do not match the data characteristics, causing the optimization to end quickly in the traditional single-optimizer mode. At this time, it is worth combining multiple optimizers to co-optimize model parameters as it may find a better evolutionary path for parameter optimization. Sadly, this kind of work has not been seen so far.

To improve this situation, we propose a dual optimizer relay mechanism. The core idea of this mechanism is to divide the optimization of model parameters into two stages. In the first stage, we used one selected optimizer to optimize parameters to the best of its ability. We then switch to the second optimizer and continue to optimize upon the optimized parameters in the first stage. Our dual optimizer relay mechanism fixes Adam as the first optimizer since it is a commonly used optimizer with good effect. We only selected the second appropriate optimizer for each dataset. Our experience shows that the optimal combination of optimizers vary for different datasets.

### Model complexity analyses

Let us now analyze the complexity of our model from the following two aspects: the parameter number and the time complexity. Although there are MLP parameters in the graph convolution layer, the embedding layer provides most trainable parameters since the attributes number in dataset is usually much larger than the embedding dimension. We denote the attributes number for users and items as *J* and *K*, respectively, and the embedding size be *d*. Then the embedding layer occupies (*J* + *K*) × *d* parameters. From the perspective of parameter number, the complexity of our model is at a low level for that the number of trainable parameters is similar to FM.

For model training, compared with previous GNN-based models such as Fi-GNN, *L*_0_-SIGN and GMCF, the difference of time complexity was mainly caused by the graph construction since the more edges, the more computations in the graph convolution layer. Given a dataset, suppose each user has *P* attributes and each item has *Q* attributes in average, then the time taken when performing one graph convolution layer using previous models is approximately *O*((*P*∗*Q* + *P*^2^ + *Q*^2^)*d*). However, the time complexity for our proposed model is approximately *O*((*P*∗*Q*)*d*). It is worth noting that usually *Q* is larger than *P*. In many cases, nearly half of the training time can be saved, which will be shown in the ablation study. We can also find that the time complexity can be reduced from approximately the power order *O*(*Q*^2^*d*) to linear order *O*(*Qd*) when *Q* is much larger than *P*, which makes the training process much faster.

## Evaluation

In this section, we first introduce the datasets, baseline methods, and experimental settings used in our experiments. Then, we investigate the performance of our proposed LightFIG compared with existing baselines to verify its effectiveness. Finally, we make further analysis of our model under different experimental settings.

### Dataset description

To evaluate the performance of our proposed method, we conduct experiments on three datasets from MovieLens 1M, Book-Crossing and Taobao, which vary in domain and size. All datasets can be accessed at GitHub (https://github.com/diweiqiang/LightFIG/tree/master/data). We summarize the statistics of them in Table 1. The ratio of attributes number of users to that of items in average for the constructed attribute graph is shown in the last column. This can be helpful in estimating the time complexity of the model. Below are descriptions of the used datasets:

• **MovieLens 1M** (Harper & Konstan, 2015): A widely adopted dataset in movie recommendation. It contains movie ratings and corresponding side information about users and movies.
• **Book-Crossing** (Ziegler et al., 2005): A dataset about users’ ratings of books. Besides, more information about the user and consumed book can be found in their attributes.
• **Taobao** (Zhou et al., 2018): A collected traffic logs of clicks on displayed advertisements showed on the shopping site of Taobao. Each data record contains a user, a displayed advertisement and other side information useful for recommendation.

In order to be consistent with previous models when preparing data samples, we keep those with ratings no less than four as positive ratings for MovieLens 1M and treat all ratings for Book-Crossing as positive ratings for that not much data is available. The same number of negative samples are chosen to pair the positive samples. To filter noisy data, we only keep users with at least 10 positive ratings for MovieLens 1M and have at least 20 positive ratings for Book-Crossing and Taobao.

**Table 1:** Dataset statistics. The *attr* refers to “attributes”.

Dataset	#Data	#User	#Item	#User attr	#Item attr	Attr ratio
MovieLens 1M	1,149,238	5,950	3,514	30	6,944	4:9.5
Book-Crossing	1,050,834	4,873	53,168	87	43,157	3:7.7
Taobao	2,599,463	4,532	371,760	36	434,254	6:6.1

For each dataset, we randomly selected 60% of the total samples as the training set, another 20% as the validation set, and the last 20% as the test set. The validation set was used to search for better parameter settings, and the test set is used to evaluate the final performance.

### Baselines

To demonstrate the effectiveness of LightFIG, we compare it with several methods as follows:

• **FM** (Rendle, 2010): A competitive model which applies a sum of pairwise dot product of features to obtain the prediction score.
• **AFM** (Xiao et al., 2017): Attention mechanism is used to adjust the weight of each interaction in FM.
• **NFM** (He & Chua, 2017): A model leverageing a MLP to learn nonlinear and high-order interaction among features.
• **W&D** (Cheng et al., 2016): It is a deep neural network joined with a linear model.
• **DeepFM** (Guo et al., 2017): DeepFM shares the feature embedding between the FM and the deep neural network.
• **AutoInt** (Song et al., 2019): It learns high-order feature interactions by applying a multi-head self-attentive neural network.
• **DGCF** (Wang et al., 2020): It considers user-item relationships at the intents level and generates disentangled representations.
• **Fi-GNN** (Li et al., 2019b): It proposes to represent the multi-field features in a graph structure for the first time.
• ***L*_0_-SIGN** (Su et al., 2021a): It detects the beneficial feature interactions *via* a graph neural network approach and *L*_0_ regularization.
• **NIA-GNN_*l*0_**: We utilize the two neighbor aggregation mechanisms proposed in NIA-GNN (Sun et al., 2020). Since the mechanisms used in NIA-GNN are for incomplete graph, we therefore apply them upon the learned graph by *L*_0_-SIGN.
• **GMCF** (Su et al., 2021b): It highlights the different impacts of attribute interactions and treats them differently when doing predictions.

### Parameter settings

We implemented our model in Pytorch. The embedding size *d* was fixed to 64 and the hidden size *h* in Eq. (5) was 4*d* for fair comparison. The coefficient *λ* for parameter regularization in Eq. (10) was set as 1 × 10^−5^. The batch size chosen was 128. In our dual optimizer relay mechanism, the optimizer for the first stage was Adam for all datasets, with learning rates denoted as *lr*_1_ set to 0.001. RMSprop was selected as the optimizer in the second stage for datasets MovieLens 1M and Book-Crossing with learning rates denoted as *lr*_2_ set to 0.0008 and 0.0005 respectively, AdaGrad was used as the optimizer in the second stage for dataset Taobao with learning rate *lr*_2_ of 0.1. For parameter settings of most baseline methods, we follow the configuration as in the article on GMCF. For NIA-GNN_*l*0_, the number of layers *k* = 2. We adopted three widely-used protocols to evaluate the quality of prediction: Area Under ROC (AUC), Logloss, and Normalized Discounted Cumulative Gain (NDCG). AUC and Logloss are widely used metric in binary classification and NDCG@k is a common metric to evaluate the top-k recommendation. We set k to 5 and 10. All experiments were repeated five times and the average results was taken.

### Performance comparison

We first compared results of all methods on the three datasets. Table 2 presents the overall performance and we have the following observations:

• FM and AFM achieve the worst performance on all three datasets, indicating that only dot product itself is not good to extract information from attribute interactions. NFM and DeepFM achieve better performance than FM and AFM by a large margin. This is because they can model more complex feature interactions through the MLP to capture nonlinear relationships. The performance of AutoInt is also good, which demonstrates the potential of the self-attention mechanism, and verifies that simply using MLP might limit the representation learning and interaction modeling.
• From the results, DGCF performs relatively poor on three datasets. This may be due to that attributes are not as decomposable as items DGCF was originally applied. NIA-GNN_*l*0_’s performance is decent, but the improvement upon *L*_0_-SIGN isn’t huge. This may be attributed to the edges in the graph, which are learned by L0-GNN, since the accuracy of these connections is not so reliable, leading to limited performance improvement. Attribute graph based methods like LightFIG and GMCF achieve better results. This provides evidence that modeling the connectivity information among attributes is beneficial to obtain better embeddings in learning the collaborative signals. GNN can learn more meaningful representations by performing message propagation on the attribute graph.
• Our proposed model consistently outperforms all baselines with respect to all measures. Regarding the *t*-test, the improvements LightFIG achieved are statistically significant with the *p*-value on all metrics less than 0.05. We attribute the performance boost to: (1) LightFIG constructs a more efficient attribute graph which gets rid of lots of redundant or even interfering edges, and focuses on mining relationships only between user attributes and item attributes. Better embedding propagation over the attribute graph and information fusion mechanism are designed to distill informative features from neighbors; (2) The dual optimizer relay mechanism upgrades the traditional single-optimizer process to a dual-optimizer process. Two different properly chosen optimizers can leverage their respective strengths and coordinate to continuously optimize model parameters.

### Ablation study

In this section, we conduct experiments to analyze different components in our model. Several variants are developed to verify the rationality of some designs. Results are illustrated in Table 3.

**Table 2:** Overall Performance Comparison. The bold indicates the best result, while the second-best performance is underlined.

Methods	MovieLens 1M				Book-Crossing				Taobao			
	AUC	Logloss	NDCG@5	NDCG@10	AUC	Logloss	NDCG@5	NDCG@10	AUC	Logloss	NDCG@5	NDCG@10
FM	0.8761	0.4409	0.8143	0.8431	0.7417	0.5771	0.7616	0.8029	0.6171	0.2375	0.0812	0.1120
AFM	0.8837	0.4323	0.8270	0.8676	0.7541	0.5686	0.7820	0.8258	0.6282	0.2205	0.0872	0.1240
NFM	0.8985	0.3996	0.8486	0.8832	0.7988	0.5432	0.7989	0.8326	0.6550	0.2122	0.0997	0.1251
W&D	0.9043	0.3878	0.8538	0.8869	0.8105	0.5366	0.8048	0.8381	0.6531	0.2124	0.0959	0.1242
DeepFM	0.9049	0.3856	0.8510	0.8848	0.8127	0.5379	0.8088	0.8400	0.6550	0.2115	0.0974	0.1243
AutoInt	0.9034	0.3883	0.8619	0.8931	0.8130	0.5355	0.8127	0.8472	0.6434	0.2146	0.0924	0.1206
DGCF	0.9011	0.3899	0.8602	0.8907	0.8109	0.5357	0.8111	0.8453	0.6388	0.2187	0.0893	0.1202
Fi-GNN	0.9063	0.3871	0.8705	0.9029	0.8136	0.5338	0.8094	0.8522	0.6462	0.2131	0.0986	0.1241
*L*_0_-SIGN	0.9072	0.3846	0.8849	0.9094	0.8163	0.5274	0.8148	0.8629	0.6547	0.2124	0.1006	0.1259
NIA-GNN_*l*0_	0.9099	0.3803	0.9021	0.9204	0.8189	0.5259	0.8322	0.8793	0.6632	0.1981	0.1083	0.1361
GMCF	0.9127	0.3789	0.9374	0.9436	0.8228	0.5233	0.8671	0.8951	0.6679	0.1960	0.1112	0.1467
LightFIG	**0.9238**	**0.3622**	**0.9457**	**0.9523**	**0.8457**	**0.5018**	**0.8870**	**0.9131**	**0.6810**	**0.1944**	**0.1253**	**0.1612**
*Improv*	1.22%	4.41%	0.89%	0.92%	2.78%	4.10%	2.29%	2.01%	1.96%	0.82%	12.7%	9.88%
*p-value*	8.4e−5	9.3e−4	2.5e−5	3.9e−5	5.7e−5	8.4e−4	2.2e−5	1.7e−5	2.9e−4	6.4e−3	3.4e−3	1.8e−3

**Figure 2:**
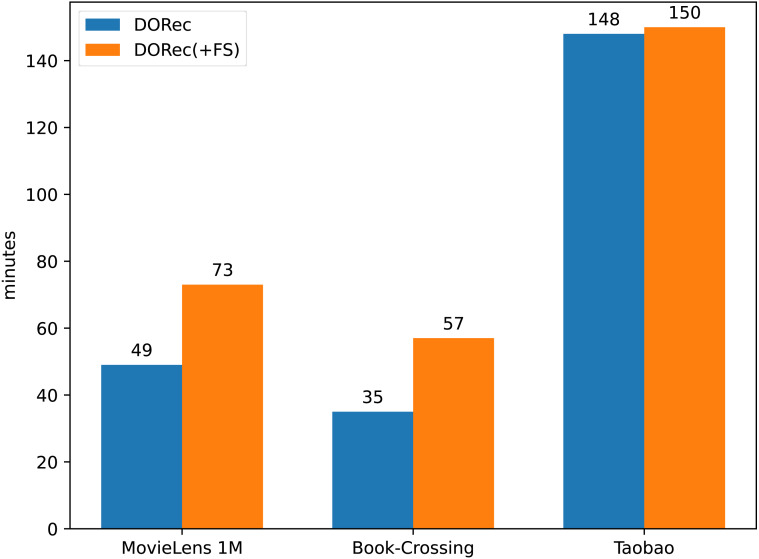
The comparison of average training time.

LightFIG(+FS) is LightFIG with additional edges between user (item) attributes. These added edges do not obviously improve performance on datasets MovieLens 1M and Book-Crossing, which means they are redundant here. Not only that, they also carry a lot of extra computations. Figure 2 demonstrates the time cost of different methods. We can see that the training time of our model is greatly reduced on both datasets MovieLens 1M and Book-Crossing. The advantage of time cost on dataset Taobao is not obvious. This is because in the first two datasets, the average attributes number of items is larger than that of users, but this is not the case in Taobao, which can be seen in the last column of Table 1. To make matters worse, these extra edges can even interfere with the model performance on dataset Taobao with a non-negligible performance drop in NDCG@10. This fully shows the necessity of removing redundant edges as done in our method.

**Table 3:** Ablation study with different variants of our model.

Methods	MovieLens 1M		Book-Crossing		Taobao	
	AUC	NDCG@10	AUC	NDCG@10	AUC	NDCG@10
LightFIG(+FS)	0.9240	0.9510	0.8462	0.9112	0.6814	0.1590
LightFIG(-D)	0.9186	0.9484	0.8406	0.9077	0.6683	0.1466
LightFIG(-G)	0.9158	0.9465	0.8227	0.8976	0.6793	0.1584
LightFIG(-CA)	0.9133	0.9454	0.8030	0.8820	0.6772	0.1593
LightFIG(-C)	0.9175	0.9482	0.8365	0.9050	0.6735	0.1581
LightFIG(-ME)	0.9225	0.9509	0.8417	0.9095	0.6818	0.1590
LightFIG(-MA)	0.9213	0.9501	0.8421	0.9076	0.6803	0.1579
LightFIG	0.9238	0.9523	0.8457	0.9131	0.6810	0.1612

LightFIG(-D) is LightFIG removing the dual optimizer relay mechanism. From the results we can address that incorporating this dual-optimizer co-optimization approach brings significant improvements in the recommendation accuracy. Besides, Our empirical experiments show that in MovieLens 1M and Book-Crossing, better results can be achieved using Adam and RMSprop. While Adam and AdaGrad is a more suitable combination on dataset Taobao, which will be further explained later.

LightFIG(-G) is LightFIG without the gating mechanism demonstrated in Eq. (8), they are directly added instead. We can see from the results that removing this mechanism causes a consistent drop in performance, which demonstrates its effectiveness. Moreover, the gating mechanism works much better on datasets MovieLens 1M and Book-Crossing than the third Taobao. We speculate this is because Taobao is more sparse, only embeddings updated through GNN structure can capture useful information for the recommendation task in this scenario.

In the message propagation part with Eq. (5), LightFIG use three cross-feature interactions to construct useful input for subsequent MLP. They are concatenation, element-wise product and element-wise addition. To test their validity, we remain only element-wise product in variant LightFIG(-CA), remain element-wise product and element-wise addition in variant LightFIG(-C). The results show that concatenation and element-wise addition can stack on top of element-wise product and gain positive benefits continuously, which proves the necessity of some artificial cross features when propagating message.

We now examine the impact of global average pooling and max pooling when integrating the attribute representations of a user (item) done in Eq. (7) (since this worked well, we did not consider other more sophisticated alternatives). LightFIG(-ME) is LightFIG without the global mean pooling and LightFIG(-MA) is LightFIG without the global max pooling. The results show that both max and mean pooling are effective, justifying their selection as the feature fusion scheme.

### Impact of high-order connectivity

We now attempt to understand whether stacking more graph convolution layers will facilitate the representation learning with information propagated from multi-hop neighbors.

We used LightFIG-2 to denote the model with two graph convolution layers, and similar notations for others. It is worth emphasizing here that our LightFIG has only one graph convolution layer. The performance is reported in Table 4. A key observation we can find is that adding more layers does not get the expected stronger performance. Instead, there is a non-negligible performance drop. Experimental results here show that messages from multi-hop neighbors help little or even harm, as the benefits of stacking more layers mainly comes from fusing information of multi-hop neighbors. Taking a random node of user attributes in the attribute graph as an example, its one-hop neighbors are nodes of item attributes and its two-hop neighbors are nodes of user attributes. This shows that for embedding representations of user attributes, the first-order interactions with item attributes are the key for recommendation, and the attribute interactions within the user attributes do not help. The same is true for item attributes. This just proves one core point of our article: the correlation between user attributes and item attributes is what we need to pay more attention to, not that within user attributes or item attributes themselves.

**Table 4:** The impact of depth in graph convolution.

Methods	MovieLens 1M		Book-Crossing		Taobao	
	AUC	NDCG@10	AUC	NDCG@10	AUC	NDCG@10
LightFIG-2	0.9187	0.9487	0.8367	0.9041	0.6736	0.1590
LightFIG-3	0.9180	0.9484	0.8362	0.9046	0.6738	0.1591
LightFIG	0.9238	0.9523	0.8457	0.9131	0.6810	0.1612

### Impact of optimizer combinations

In this section, we conduct experiments to analyze the influence of different optimizer combinations. We developed several variants to better understand their effectiveness. It is worth noting that the dual optimizer relay mechanism fixes Adam as the first optimizer and only select the appropriate second optimizer for different datasets.

**Figure 3:**
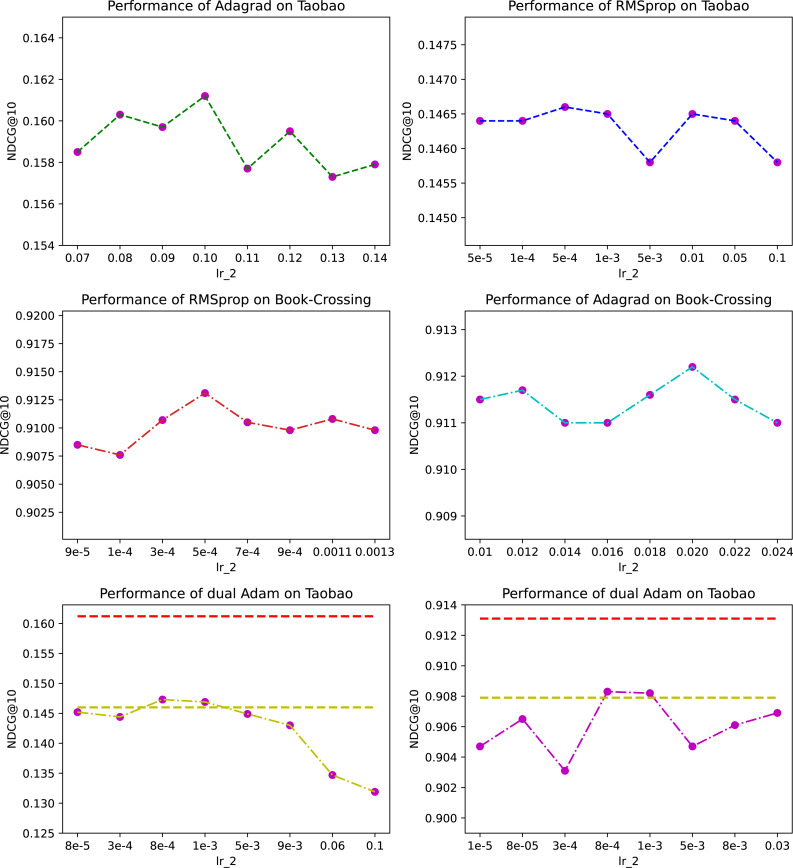
The effect of different optimizer combinations.

Let’s first take the Taobao dataset as an example. The first row in Fig. 3 shows the prediction performance with Adagrad and RMSprop as the second optimizer respectively and *x*-axis represents different learning rate of the second optimizer. The sub-figure about Adagrad shows the dynamics around the optimal value we searched for, and note that this optimizer was also finally adopted by LightFIG for this dataset. The sub-figure about RMSprop shows what performance looks like on part of nodes we have searched. Clearly, despite our extensive search on RMSprop, Adagrad outperforms RMSprop with a large margin. This shows that different optimizer combinations can have a significant impact on performance.

Different combination of optimizers can sometimes achieve the same effect. We now look at the influence on dataset Book-Crossing. The second row in Fig. 3 shows the results with RMSprop and Adagrad as the second optimizer respectively. We can find that the best performance they can achieve is very close. This shows that the optimal optimizer combination may not be unique.

Usually two optimizers of the same type do not achieve better results, on the contrary they are likely to cause performance degradation. Let’s take Adam’s performance on the Taobao dataset as an example. The results are shown in the last row of Fig. 3. We did a wide-ranging search on the learning rate to ensure the reliability of results and the graph shows part of values we have searched for. The red dashed line at the top is the optimal value LightFIG has achieved, and the yellow dashed line is the optimal value that can be achieved using a single Adam optimizer. The results show that using the same type of optimizer does not help optimize parameters any better, but is more likely to rapidly deteriorate the learned values. It is difficult to find a better optimization path from the perspective of the same type of optimizer. We only show results on the Taobao dataset, but there are similar conclusions on other datasets.

We did not just test the combined effects of the above three optimizers, but they did not work well, and considering the limitation of space, we will not show them here.

## Conclusion

In this work, our improvements are mainly carried out in two aspects. The first one is that we propose to simplify the unnecessarily complicated attribute graph for collaborative filtering and focusing on mining relationships between user attributes and item attributes. By constructing an efficient attribute graph and better embedding propagation mechanism, we can not only save lots of training time but may also boost the recommendation performance. The second one is that we propose a dual optimizer relay mechanism, which changes the traditional single-optimizer pattern and employs two different types of optimizers to coordinate optimizing network parameters. The extensive experiments on three real-world datasets have demonstrated the superiority of our proposed LightFIG over the state-of-the-art methods.

For future work, we are interested in designing a simple yet effective method to calculate attribute correlations and provide a more accurate basis for whether to link, to change the situation of blindly linking attributes due to the less of supervision signal for associations between user and item attributes.

## Supplemental Information

10.7717/peerj-cs.1019/supp-1 Supplemental Information 1 Python code

## Additional Information and Declarations

### Data Availability

The following information was supplied regarding data availability: The code is available in the Supplemental Files. The data is available at GitHub: https://github.com/diweiqiang/LightFIG/tree/master/data.
